# Editorial: Insights in thrombosis: 2021

**DOI:** 10.3389/fcvm.2022.1021626

**Published:** 2022-09-21

**Authors:** Hugo ten Cate

**Affiliations:** Internal Medicine, Maastricht University Medical Centre, Maastricht, Netherlands

**Keywords:** thrombosis, diagnostic test, COVID-19, pulmonary embolism, protein C activator

A topic with a concise, but rather non-specific title “Insights” is deemed to attract submissions with a broad coverage. Indeed, the 6 articles that were accepted cover topics from the broad spectrum of thrombosis and hemostasis. This is exciting as it shows how diverse our field is, as it addresses topics that one is not even considering on a daily basis.

Thus, etiology, diagnostics and management are covered in this concise series.

A timely narrative review paper discusses issues related to the histology of thrombosis in lungs from subjects that contracted COVID-19 (Spier and Evans). Both 2D and 3D reconstructions of microvascular thromboinflammation give very sensitive images of the key players involved in this COVID-19 pulmonary manifestations. Insight in key players like endothelial cells, mononuclear cells and megakaryocytes/platelets provides a basis for understanding this disease, possibly providing a clue to improved treatment. As it is evident that this virus tends to recur, lessons from histology are needed to prepare for improved imaging and management, in a next pandemic.

While pulmonary thrombosis and pulmonary embolism (PE) coincide in COVID-19, PE itself is a common manifestation of venous thromboembolism (VTE) that occurs either idiopathic (unprovoked) or secondary to other diseases (e.g., cancer) or risk factors (e.g., oral contraceptives). In a report from an ongoing large registry in China, the authors provide detailed data on about 10% of the subjects with PE that also suffered from syncope, a known sign of poor prognosis. Not surprisingly, in their series syncope was associated with hemodynamic instability and a larger need for thrombolysis. However, syncope as such was not associated with in-hospital mortality; a principal component analysis-based cluster analysis yielded 4 different syncope+ PE phenotypes of which one particular phenotype, that was characterized by frequent CVD, recent surgery/trauma, less frequent malignancy and relatively high pulse and respiratory rates, was associated with highest all cause and PE related mortality (Zhang S. et al). Such phenotyping of subjects suffering from PE and syncope, can possibly add in better selection of highest risk patients requiring thrombolysis.

Intra-arterial thrombolysis as management for the hypothenar hammer syndrome (HHS), a rare trauma related hypothenar ischemic manifestation, was studied in this systematic review of the literature (Jud et al). HHS is a syndrome particularly encountered in persons that professionally or accidently use their hand as hammer or whose hand(s) are exposed to vibrating tools. Such (repeated) traumata may elicit thrombosis that may embolize into digital arteries, producing ischemia. Although various empiric management strategies have been applied, in absence of randomized trials, intra-arterial thrombolysis was reported in 16 papers, involving in total 43 patients. Local thrombolysis in combination with systemic heparinization was associated with a significantly improved reperfusion as compared to thrombolysis alone with a median duration of thrombolysis of 17 h, where treatment for longer than 24 h did not provide extra benefit. In these subjects bleeding was not reported. The authors conclude that intra-arterial thrombolysis with heparin is a potentially effective and safe strategy in patients with HHS.

Two contributions address laboratory diagnostic issues. Reda, Rühl, et al. assessed diagnostic accuracy of protein C deficiency testing comparing Protac, a snake venom derived protein C activator, with thrombin-thrombomodulin complex; the latter turned out to be a superior activator of protein C under the test conditions, providing 100% correctly identified protein C deficiency results. In order to directly probe activated protein C in plasma the authors also made use of an oligonucleotide-based enzyme capture assay using an exosite directed aptamer. These data collectively provide a good basis for accurate protein C testing in case of suspected deficiency of this natural anticoagulant protein.

In a second, different study from the same institution, Reda, Serra, et al. retrospectively studied the impact of oral anticoagulation for venous thromboembolism on plasma d-dimer levels during anticoagulation. As compared to vitamin K antagonists, the use of direct oral anticoagulants was associated with statistically higher d-dimer levels, for rivaroxaban, apixaban and dabigatran. The recommendation of the authors is that one should be cautious to extrapolate algorithms, that make use of d-dimer, developed in patients on VKA, to patients on DOAC, for the prediction of (recurrent) thrombosis. Moreover, the data suggest that the suppressive effect of DOAC on coagulation is on average less profound than of INR guided VKA treatment. The causes of such differences may include interindividual variation in pharmacokinetics of DOAC, but also more trivial matters like adherence to medication and quality of VKA management.

Finally, Zhang W. et al. provide a very interesting review on a topic that has not gained wide exposure yet, i.e., the lymphatic hemostatic mechanism in relation to health and disease. They provide an overview of anatomy, physiology and composition of lymphatic hemostatic proteins and cells expressing tissue factor or binding factor X. Outlining the differences in ratio between pro- and anticoagulant molecules in relation to lymphatic flow, the specific characteristics of lymphatic hemostasis emerge and this provides a basis for a better understanding of lymphatic thrombosis, that may occur in conditions like sepsis and cancer. They do not address the issue of management of lymphatic thrombosis for which one assumes that treatment of the underlying disorder is key, while anticoagulants that impact the circulating blood most likely also affect lymphatic hemostasis.

## Author contributions

The author confirms being the sole contributor of this work and has approved it for publication.

## Conflict of interest

The author declares that the research was conducted in the absence of any commercial or financial relationships that could be construed as a potential conflict of interest.

## Publisher's note

All claims expressed in this article are solely those of the authors and do not necessarily represent those of their affiliated organizations, or those of the publisher, the editors and the reviewers. Any product that may be evaluated in this article, or claim that may be made by its manufacturer, is not guaranteed or endorsed by the publisher.

